# Differences in MR perfusion of malignant and benign cardiac tumors

**DOI:** 10.1186/1532-429X-11-S1-P164

**Published:** 2009-01-28

**Authors:** Kerstin U Bauner, Steven Sourbron, Michael Schmoeckel, Maximilian F Reiser, Armin M Huber

**Affiliations:** Ludwig-Maximilian-University Munich, Campus Grosshadern, Munich, Germany

**Keywords:** Rhabdomyosarcoma, Angiosarcoma, Maximum Slope, Cardiac Tumor, Dynamic Magnetic Resonance Imaging

## Introduction

Primary cardiac tumors are a rare disease while secondary cardiac neoplasms are found more often. The localisation of the tumor, the patient's age and if present, primary malignancy or systemic disease, may give hints to the characterization of a particular cardiac neoplasm. Unenhanced T1- and T2- weighted MR-imaging and fat and suppression techniques allow for comprehensive tissue characterisation. However, additional information can be derived from first pass perfusion imaging and sometimes diagnosis can be made without biopsy and surgery.

## Purpose

To determine whether dynamic contrast material-enhanced magnetic resonance (MR) imaging with use of kinetic parameters reveals statistically significant differences between benign and malignant cardiac tumors.

## Methods

This study involved 23 patients with cardiac tumors (Myxoma (n = 7), Lipomatous hypertrophy of the interatrial septum (n = 2), Fibroma (n = 1), Rhabdomyoma (n = 2), Angiomyolipoma (n = 1), Angiosarcoma (n = 4), Rhabdomyosarcoma (n = 1), Hemeangiomyosarcoma (n = 1), Myoliposarcoma (n = 2), Lymphoma (n = 1), Metastasis (n = 1). The patients were examined with a T1 weighted turboFLASH sequence. The data were transferred to an external workstation and postprocessing was performed by using software written in-house in IDL 5.4. Contrast enhancement ratios, maximum slope of contrast enhancement ratio curve (% / sec) and the area under the contrast enhancement ratio curve (% * sec) were calculated. Statistical analysis were performed with the unpaired t-test. P values less than 0.05 were considered statistically significant. In addition sensitivities and specificities to differentiate benign from malignant masses were calculated for each parameter and receiver-operator-characteristics (ROC) were assessed.

## Results

**Figure 1:**
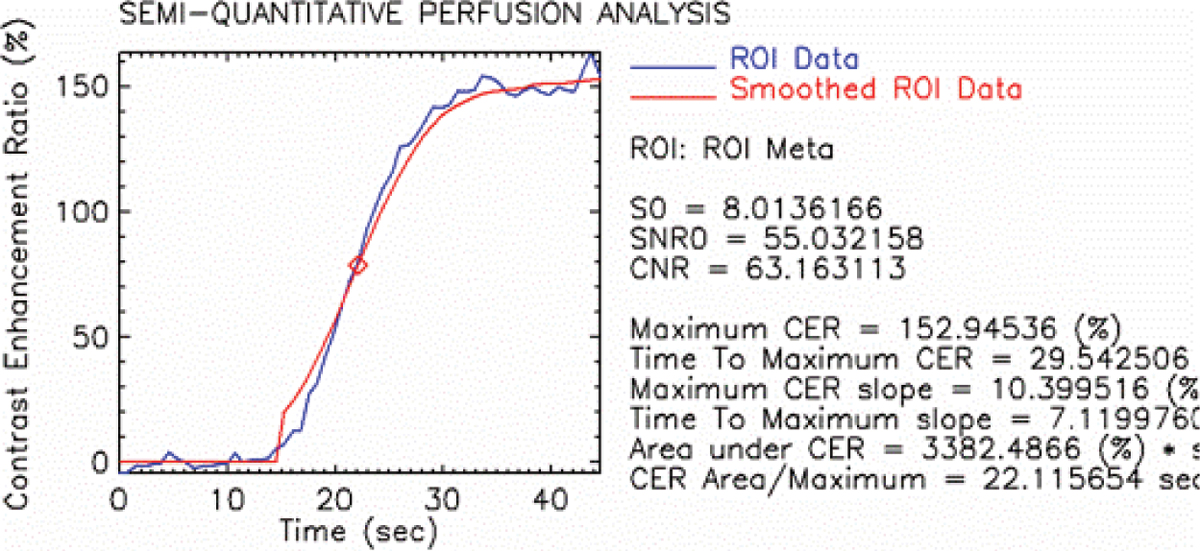
Figure 1

**Figure 2:**
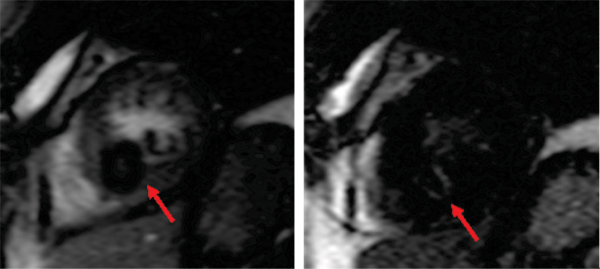
Figure 2

**Figure 3:**
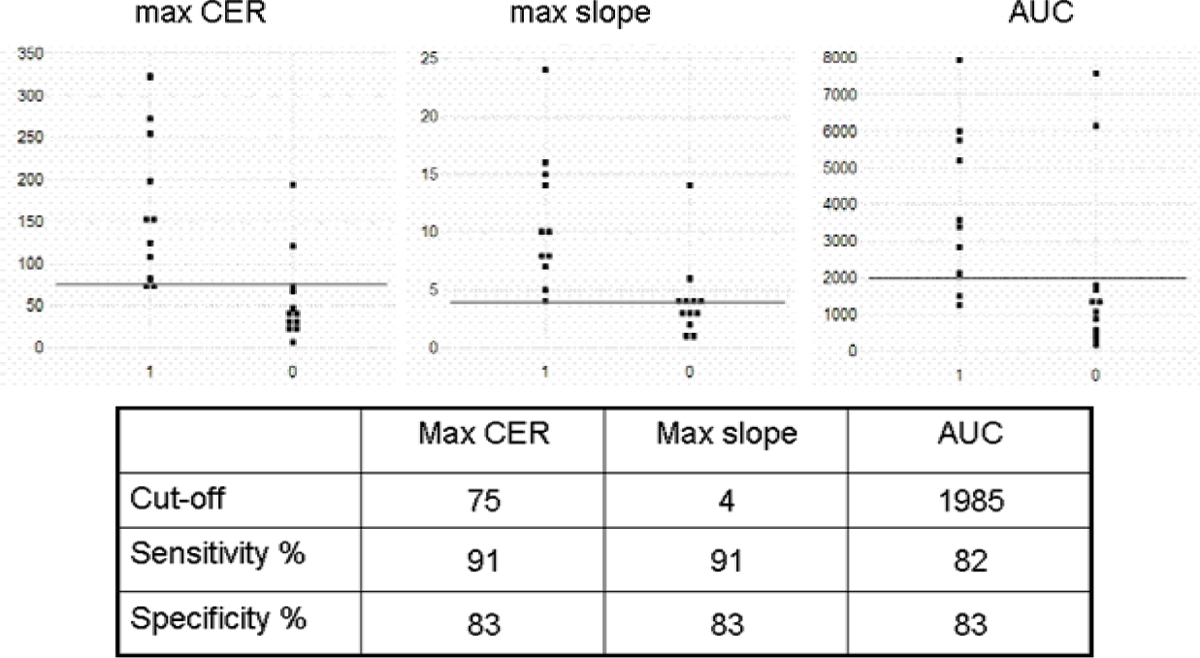
Figure 3

The contrast enhancement ratio (CER %) was significantly higher (p = 0.0035) in malignant cardiac tumors (153.9 ± 82.5) compared to benign lesions (57.8 ± 52.5), as was the maximum slope of CER (% / sec) (p = 0.01; 10.5 ± 5.9 vs. 4.7 ± 3.9). The values for the area under the curve (% * sec) were significantly higher (p = 0.0011) in malignant cardiac lesions (4204.5 ± 2122.3) in comparison to benign lesions (1253.2 ± 1135.2). The calculated sensitivity and specificity for the CER, the maximum slope of CER and the area under the curve resulted in 91% and 83%, 91% and 83% and 82% and 82% respectively with a cut-off value of 75%, 4%/sec and 1985%*sec. The ROC analysis revealed identical values for the areas under the ROC curves for CER and the maximum slope of CER (AUC = 0.913). The ROC for the area under the contrast enhancement ratio curve was 0.803. See Figures 1, 2, 3.

## Conclusion

Dynamic MR imaging delineates significant kinetic differences in perfusion between malignant and benign cardiac tumors. Semiquantitative assessment of perfusion parameters can help to discriminate these by determination of contrast enhancement ratios, calculation of the maximum slope of the CER curve and the area under the CER-curve.

